# Neutrophils as a Source of Chitinases and Chitinase-Like Proteins in Type 2 Diabetes

**DOI:** 10.1371/journal.pone.0141730

**Published:** 2015-10-30

**Authors:** Ewa Żurawska-Płaksej, Agnieszka Ługowska, Katarzyna Hetmańczyk, Maria Knapik-Kordecka, Agnieszka Piwowar

**Affiliations:** 1 Department of Pharmaceutical Biochemistry, Wroclaw Medical University, Wroclaw, Poland; 2 Department of Genetics, Institute of Psychiatry and Neurology in Warsaw, Warsaw, Poland; 3 Department of Angiology, Hypertension and Diabetology, Wroclaw Medical University, Wroclaw, Poland; 4 Department of Toxicology, Wroclaw Medical University, Wroclaw, Poland; Université PARIS- DIDEROT (7), FRANCE

## Abstract

**Purpose:**

The pathophysiological role of human chitinases and chitinase-like proteins (CLPs) is not fully understood. We aimed to determine the levels of neutrophil-derived chitotriosidase (CHIT1), acidic mammalian chitinase (AMCase) and chitinase 3-like protein 1 (YKL-40) in patients with type 2 diabetes (T2D) and verify their association with metabolic and clinical conditions of these patients.

**Methods:**

Neutrophils were obtained from the whole blood by gradient density centrifugation from 94 T2D patients and 40 control subjects. The activities of CHIT1 and AMCase as well as leukocyte elastase (LE) were measured fluorometrically and concentration of YKL-40 immunoenzymatically. Also, routine laboratory parameters in serum/plasma were determined by standard methods.

**Results:**

The levels of all three examined proteins were about 2-times higher in diabetic patients in comparison to control subjects. They were significantly correlated with the activity of LE and increased progressively across tertiles of LE activity. Moreover, the activities of CHIT1 and AMCase were significantly correlated with each other. Metabolic compensation of diabetes did not influence the levels of these proteins. In the subgroup of patients with inflammatory evidence only YKL-40 concentration was significantly higher compared to those without inflammation. The highest levels of all three proteins were observed in patients with macroangiopathies. Insulin therapy was associated with lower levels of examined proteins.

**Conclusions:**

We revealed that neutrophils may be an important source of the increased levels of chitinases and CLPs in T2D, and these proteins may participate in inflammatory mechanisms in the course of the disease and consequent development of diabetic angiopathies.

## Introduction

The evolutionarily conserved glycosyl hydrolase 18 family (GH18) contains a few chitinases and chitinase-like proteins (CLPs) that are expressed in mammals. Intriguingly, although endogenous chitin has not been identified in humans, two enzymatically active chitinases, chitotriosidase (CHIT1) and acidic mammalian chitinase (AMCase), as well as at least three proteins with chitin-binding ability, but without chitinase activity, such as chitinase 3-like protein 1 (termed as YKL- 40 or human cartilage glycoprotein-39), have been found in different human cells [1].

Chitotriosidase is produced mainly by monocyte-derived macrophages and is an established marker of macrophage accumulation in lysosomal storage diseases (especially in Gaucher disease) [2]. It may also be synthesized by neutrophils and released from the specific granules upon stimulation [3]. Acidic mammalian chitinase is produced mainly by macrophages and lung epithelial cells at sites of Th2-mediated inflammation, associated with eosinophil recruitment [4]. Presence of this enzyme in neutrophils has not yet been described. YKL-40 is known to be expressed in macrophages (but not in monocytes), as well as in neutrophils, chondrocytes, and tumor cells. It has been considered as a macrophage differentiation marker, which may contribute to the cellular response induced by proinflammatory cytokines, such as interleukin-1 (IL-1) and tumor necrosis factor alpha (TNF-α) and the migration factor for vascular smooth muscle cells [5]. Moreover, YKL-40 stimulates the proliferation of human connective tissue cells (such as fibroblasts) [6,7].

Currently, the exact physiological role of proteins from GH18 in humans is not fully understood. One hypothesis assumes that active chitinases are induced at sites of infection as part of an innate defense against chitin-containing pathogens, while chitinase-like proteins are involved in signaling pathways during cell proliferation and differentiation [1,8]. An increasing number of investigations have provided evidence that the levels of these proteins are increased in a variety of diseases characterized by chronic inflammation and tissue remodeling. For example, it was revealed that increased plasma levels of CHIT1 and YKL-40, derived from macrophages, are associated with atherosclerotic plaque formation [9–11].

Diabetes mellitus type 2 (T2D) is a metabolic disease associated with obesity and consequent insulin resistance, which induce the inflammatory signaling cascade leading further to progression of metabolic disturbances and development of vascular late complications [12,13]. Macrophages are the primary cell type in the inflammatory response in T2D, although engagement of neutrophils (also called polymorphonuclear leukocytes, PMNs) is also important. Neutrophils are recruited to adipose tissue and endothelium, where they release enzymes and various pro-inflammatory mediators, which are important factors in vascular damage in diabetes [14]. There are many reports about functional impairment of neutrophils under prolonged hyperglycemia conditions [15]. However, data about the enzymatic profile of diabetic neutrophils are still ambiguous. A recent study showed involvement of neutrophils in the pathogenesis of insulin resistance via the proteolytic action of leukocyte elastase (LE) [16]. In our previous work we also observed increased activity of various neutrophilic enzymes, including LE, in patients with T2D [17]. Presence or activity of proteins from GH18 derived from neutrophils of diabetic patients has not yet been examined.

The aim of this study was to evaluate levels of selected chitinases and CLPs from polymorphonuclear leukocytes, both in healthy individuals and in patients with type 2 diabetes, as well as to evaluate whether the level of these proteins may be associated with insufficient diabetes control and vascular complications.

## Materials and Methods

### Study population

Ninety-four patients with type 2 diabetes, treated in the Clinic of Angiology, Hypertension and Diabetology of Wroclaw Medical University, and forty adults with no abnormalities in carbohydrate metabolism and no inflammatory states, as determined by a routine medical check-up, were recruited to the study. Control group subjects were matched for age, sex and BMI values with T2D patients. Diabetic patients had routine biochemical parameters, such as glucose, glycated hemoglobin (HbA1c), total cholesterol and its fraction, triglycerides (TG), total white blood cell (WBC) count and C-reactive protein concentration (CRP) measured upon admission to the hospital. All participants gave written informed consents to participate in the study.

### Cell purification

Venous blood was collected after an overnight fast in standard vacuum tubes with heparin (Sarstedt AG&Co, Germany). Neutrophils were isolated immediately using Gradisol G (AquaMed, Poland), according to the procedure described previously [18]. The isolated cells were suspended in 1 mL PBS, counted under a microscope using a Bürker chamber and stored at -80°C until assayed. The high purity (93%) of the obtained neutrophil suspension was confirmed by histochemical staining. The Local Bioethics Committee of Wroclaw Medical University specifically approved this entire study.

### Protein assay and genotyping

Before the quantitative measurements of proteins from the GH18 family, neutrophils were disrupted by a triple freezing-thawing cycle to release the intracellular content and then centrifuged (250 g for 10 min at 4°C). In the obtained supernatant enzymatic activity of CHIT1 and AMCase as well as concentration of YKL-40 were estimated. CHIT1 and AMCase activities were measured using fluorogenic substrates 4-methylumbelliferyl-β-N-N'-N"-triacetylchitotriose and 4-methylumbelliferyl-β-N-N'-diacetylchitobiose (Sigma Chemical Co, USA) in citrate-phosphate buffers with different pH and ionic strength according to methods described previously by Hollak [19] and Boot [20] and modified according to our own kinetic studies (data not shown). Briefly, to measure chitinolytic activity derived mainly from CHIT1, 5 μL of samples were incubated with 100 μL of 0.022 mM substrate solutions in 100 mM citric acid and 200 mM sodium phosphate buffer (pH 5.5). Similarly, to measure chitinolytic activity derived mainly from AMCase, 5 μL of samples were incubated with 100 μL 0.022 mM substrate solutions in 200 mM sodium citrate, 200 mM sodium phosphate buffer (pH 4.3). After 15 min incubation at 37°C, both reactions were stopped with the addition of 2 ml glycine-NaOH buffer (pH 10.6). Fluorescence of liberated products was measured at excitation and emission wavelengths of 365 nm and 445 nm, respectively (Perkin Elmer LS 50B, USA). The enzyme activities were calculated using standard calibration curves, which were constructed by plotting the fluorescence readings against different concentrations of 4-methylumbelliferone (Sigma Chemical Co, USA) in the appropriate reaction buffers and expressed as nanomoles of converted substrate per 1 hour at the reaction conditions per 1x10^6^ neutrophils within the sample. Moreover, activity of LE was measured using the fluorogenic substrate MeOSuc-Ala-Ala-Pro-Val-NMec according to Barrett [21] as described previously [17] and expressed as nanomoles of converted substrate per 1 minute at the reaction conditions per 1x10^6^ neutrophils within the sample. All measurements were performed in duplicate or triplicate. Tertiles of LE activity (T1-T3) were calculated and patients were divided into three subgroups according to values of increasing LE activity: tertile 1 (T1) included values of LE activity ≤ 62.01 nmol/min/10^6^ cells, tertile 2 (T2) > 62.01 and ≤ 133.14 nmol/min/10^6^ cells, and tertile 3 (T3) > 133.14 nmol/min/10^6^ cells. Concentration of YKL-40 was measured using the MicroVue immunoenzymatic test (Quidel, USA) and expressed in ng/10^6^ cells. The genetic deficiency of CHIT1, reflected by zero activity of this enzyme, was confirmed by detection of 24-bp duplication in exon 10 of the *CHIT1* gene as described by Boot et al. [22] in genomic DNA extracted from neutrophils (GeneMATRIX Tissue DNA Purification Kit, EURx, Poland).

### Criteria of patients'assignment to subgroups

Achievement of treatment targets, recommended by current clinical practice guidelines for management of diabetes in Poland, was the basis to assign patients to subgroups: a) patients with good and poor glycemic control (according to level of glycated hemoglobin, HbA1c), b) patients with good and poor lipid control (according to concentration of total cholesterol, low- and high-density lipoprotein fractions and triglycerides), c) patients with good and poor blood pressure control (according to values of systolic and diastolic blood pressure) [23]. Additionally, patients were assigned to subgroups based on: d) concentration of C-reactive protein (into patients with and without inflammatory states), e) presence of diabetic angiopathies (into patients with microangiopathies, macroangiopathies and both micro- and macroangiopathies) and f) received hypoglycemic treatment (into patients treated with insulin alone, treated only with oral antidiabetic agents (OAD), mainly metformin, and treated with insulin and OAD).

### Statistical analysis

The statistical analysis was performed using Statistica PL for Windows (version 10.0). Data are expressed as mean ± standard deviation. A nonparametric Mann-Whitney *U* test was used for comparison between patients and control subjects. Spearman rank correlations were used to test the mutual relationships of examined parameters. Differences between examined parameters in subgroups of patients divided according to various criteria were evaluated by the Mann-Whitney *U* test or analysis of variance (ANOVA) followed by the multiple comparison post-hoc Fisher test. A *p* value below 0.05 was considered as statistically significant.

## Results

**Fig 1:**
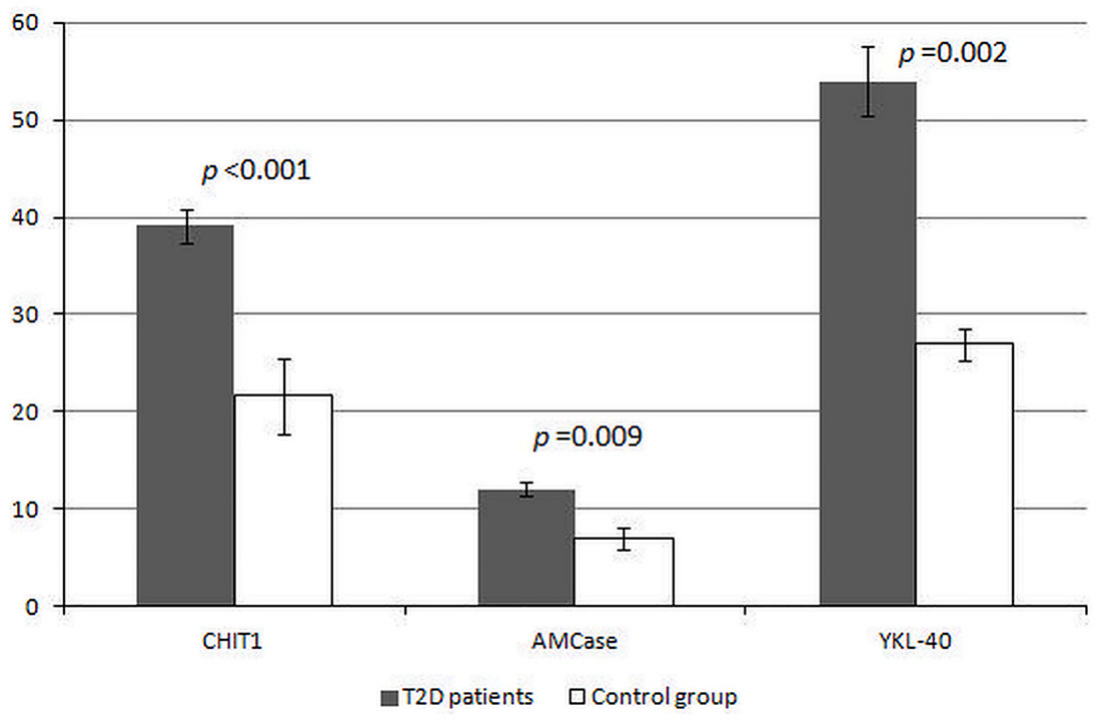
Levels of proteins from family 18 of glycosyl hydrolase in neutrophils of patients with type 2 diabetes and control subjects. CHIT1—chitotriosidase activity expressed in nmol/h/10^6^ cells, AMCase—acidic mammalianchitinase activity expressed in nmol/h/10^6^ cells, YKL-40—chitinase 3-like protein 1 concentration expressed in ng/10^6^ cells, T2D—type 2 diabetes; Data are presented as mean values ± standard error. On the y-axis appropriate units reflecting levels of examined proteins are indicated. The level of statistical significance of differences (*p* value) observed between T2D patients and the control group was evaluated by Mann-Whitney *U* test.

After laboratory measurements 13 subjects were excluded from further analysis as they showed activity of CHIT1 and/or AMCase at zero level. Finally, the examined population consisted of 85 patients with type 2 diabetes mellitus and 36 control subjects. Levels of chitotriosidase, acidic mammalian chitinase and YKL-40 in neutrophils of patients and control subjects are presented in Fig 1. All examined proteins were about 2-times higher in diabetic subjects than in the control group and all differences between these groups were statistically significant. Moreover, activity of LE in diabetic subjects was about 6-times higher in comparison to controls (131.54 vs. 21.95 nmol/min/10^6^ cells). Spearman correlation analysis revealed that examined GH18 proteins derived from diabetic neutrophils were significantly correlated with activity of LE and the strongest relationship was showed by CHIT1. Moreover, CHIT1 activity was closely associated with AMCase activity, but not with YKL-40 (Table 1).

**Table 1:** Correlations of proteins from family 18 of glycosyl hydrolase (CHIT1, AMCase and YKL-40) and leukocyte elastase in neutrophils of patients with type 2 diabetes.

Activity/concentration	CHIT1	AMCase	YKL-40
CHIT1 (nmol/h/10^6^ cells)	-	0.59***	0.23^ns^
AMCase (nmol/h/10^6^ cells)	0.59***	-	0.14^ns^
YKL-40 (ng/10^6^ cells)	0.23^ns^	0.14^ns^	-
LE (nmol/min/10^6^ cells)	0.40***	0.25*	0.33***

CHIT1—chitotriosidase, AMCase—acidic mammalian chitinase,YKL-40—chitinase 3-like protein 1, LE—leukocyte elastase, statistical significance:

***—*p* <0.001,

*—*p* <0.05,

ns—not significant.

**Fig 2:**
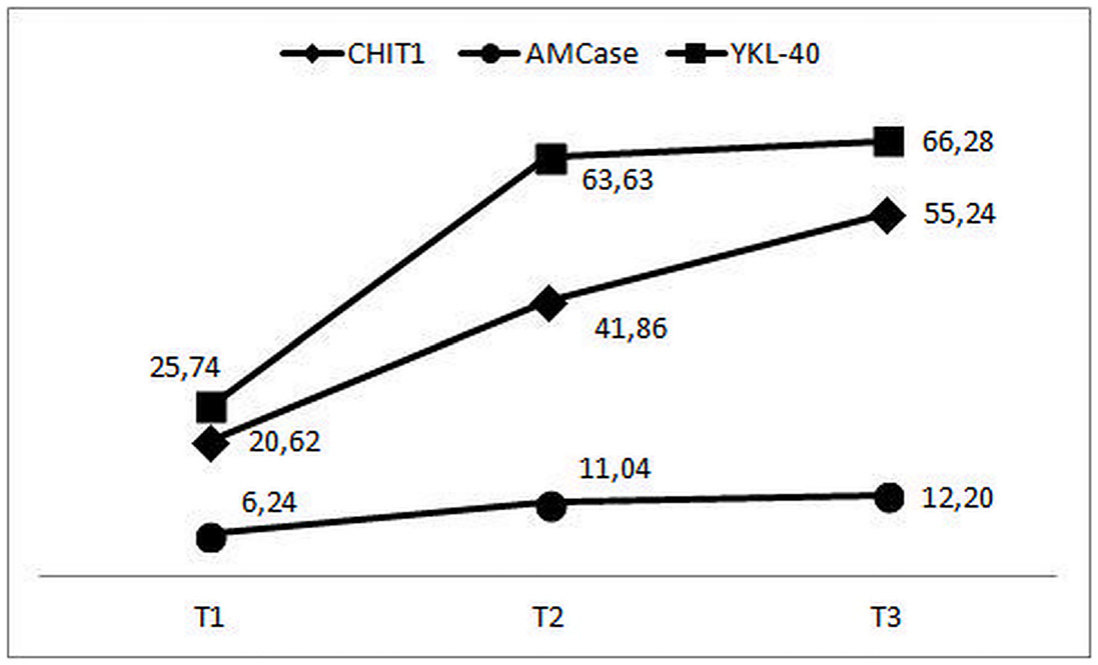
Levels of proteins from family 18 of glycosyl hydrolase in neutrophils of patients with type 2 diabetes in tertiles of increasing activity of leukocyte elastase. CHIT1—chitotriosidase activity expressed in nmol/h/10^6^ cells, AMCase—acidic mammalian chitinase activity expressed in nmol/h/10^6^ cells,YKL-40—chitinase 3-like protein 1 concentration expressed in ng/10^6^ cells,T1-T3—subgroups of patients divided according to tertiles of increasing activity of leukocyte elastase in neutrophils (for details see Material and methods section). On the y-axis appropriate units reflecting levels of examined proteins are indicated.

With regard to obtained results, we analyzed variability of levels of CHIT1, AMCase and YKL-40 in the tertiles of increasing LE activity in neutrophils (Fig 2). We observed a progressive increase in levels of all examined proteins with increasing activity of LE. However, the most evident differences were found between T1 and T3 (for CHIT1 p = 0.005, for AMCase p = 0.019, for YKL-40 p<0.001). All differences between T1 and T2 were also significant (for CHIT1 p = 0.008, for AMCase p = 0.012, for YKL-40 p = 0.002). Differences between T2 and T3 were not significant, but the most proportional trend was observed for CHIT1. The smallest degree of changes was observed for AMCase.

Levels of CHIT1, AMCase and YKL-40 in subgroups of patients divided according to different criteria (as described in Material and methods section) are presented in Table 2. Metabolic compensation of diabetes (estimated by glycemic, lipid and blood pressure control) did not significantly influence the levels of these proteins in neutrophils, but we observed slightly higher values of them in subgroups of poorly controlled patients. Enzymatic activities of CHIT1 and AMCase were also only slightly increased in patients with inflammatory states in comparison to those without inflammation. Instead, YKL-40 concentration was significantly, 1.7-times higher in patients with increased CRP concentration. Levels of examined proteins were also generally higher in patients with macroangiopathies when compared to those with microangiopathies, being the most differentiative for CHIT1 (p<0.001). Moreover, patients treated with insulin alone had a lower level of CHIT1, AMCase and YKL-40 in neutrophils than those treated with OAD. However, significant differences between all subgroups were observed only for CHIT1 activity.

**Table 2:** Levels of proteins from family 18 of glycosyl hydrolase in neutrophils of patients with type 2 diabetes divided into different subgroups according to clinical recommendations of Polish Diabetes Association.

Subgroups		CHIT1 [nmol/h/10^6^cells]	AMCase [nmol/h/10^6^cells]	YKL-40 [ng/10^6^cells]
**Glycemic control**	good (n = 44)	34.72 ± 16.07	10.39 ± 3.98	50.69 ± 24.35
	poor (n = 41)	44.91 ± 20.13	13.92 ± 5.77	57.53 ± 27.09
**Lipid control**	good (n = 14)	33.17 ± 16.79	10.63± 4.08	38.35 ± 17.65
	poor (n = 71)	40.91 ± 18.12	12.39 ± 5.43	54.76 ± 25.48
**Blood pressure control**	good (n = 49)	35.06 ±16.94	11.50 ± 5.13	51.14 ± 25.36
	poor (n = 36)	44.62 ± 20.02	12.90 ± 5.48	57.50 ± 27.08
**Presence of inflammation**	no (n = 64)	37.07 ± 17.36	11.90 ± 4.78	46.31 ± 21.15
	yes (n = 21)	45.33 ± 21.12	12.70 ± 5.04	77.39 ± 32.44 ^a^
**Diabetic angiopathies**	micro- (n = 13)	21.50 ± 13.71	7.12 ± 4.14	44.06 ± 20.51
	macro- (n = 38)	46.51 ± 21.32^b1^	13.29 ± 5.41 ^c1^	59.81 ± 25.67
	micro- and macro- (n = 34)	37.57 ± 16.93 ^b2^	12.67 ± 5.14 ^c2^	51.28 ± 22.28
**Treatment**	insulin (n = 15)	18.31 ± 8.52	8.36 ± 3.96	41.30 ± 19.23
	OAD (n = 31)	58.49 ± 24.87 ^d1^	14.55 ± 6.38 ^e^	58.87 ± 25.68
	insulin with OAD (n = 39)	31.70 ± 11.19 ^d2^,^d3^	11.59 ± 5.79	54.99 ± 22.1

CHIT1—chitotriosidase activity, AMCase—acidic mammalian chitinase activity, YKL-40—chitinase 3-like protein 1 concentration,

OAD—oral antidiabetic agents. Data are presented as mean ± standard deviation. Criteria of patients' division are detailed in the Material and methods section. Statistically significant differences (*p* value) between subgroups of patients:

^a^ without inflammation vs. with inflammation *p* = 0.011.

^b1^ micro- vs. macroangiopathies *p*<0.001.

^b2^ micro- vs. micro- and macroangiopathies *p* = 0.003.

^c1^ micro- vs. macroangiopathies *p* = 0.038.

^c2^ micro- vs. micro- and macroangiopathies *p* = 0.048.

^d1^ insulin vs. OAD *p*<0.001.

^d2^ insulin vs. insulin with OAD *p*<0.001.

^d3^ OAD vs. insulin with OAD *p* = 0.004.

^e^ insulin vs. OAD *p* = 0.005.

## Discussion

Recently there has been increasing interest in studying the pathophysiological role of GH18 members in humans. Potential involvement of these proteins in diabetes type 2 and pathological conditions associated with the disease have been partially described in single studies. Serum CHIT1 activity is reported to predict endothelial dysfunction in patients with newly diagnosed, untreated and uncomplicated T2D, while plasma YKL-40 is related with insulin resistance [24,25]. Our latest study confirmed increased plasma levels of CHIT1 and YKL-40 also in patients with long-lasting T2D [26]. However, it has not been determined whether polymorphonuclear neutrophils may be a source of these proteins in diabetes. It was previously indicated that both CHIT1 and YKL-40 are stored in specific granules of neutrophils in healthy individuals, but there is no information about AMCase, and it is not known how metabolic conditions of diabetes may influence content of these proteins in neutrophilic granules [3,27]. In the present study we observed the simultaneous, about twofold increase of all three proteins from GH18, that is CHIT1, AMCase and YKL-40, in PMNs of diabetic patients, when compared to healthy individuals. Our observations complement the insufficiency of knowledge about sources of GH18 proteins in diabetes type 2 and participation of these neutrophil-derived GH18 proteins in pathological conditions of the disease.

The increased chitinolytic activity revealed in our research, deriving mostly from CHIT1 and to a lesser degree from AMCase, is quite inexplicable in view of the lack of natural substrates for chitinases in humans. It is mentioned that these enzymes may be involved in inflammatory pathways and linked processes, but their role in diabetes remains unexplained [1,28,29]. Some authors have reported an association of increased CHIT1 activity with impaired glucose tolerance or insulin resistance, but without molecular insight [30,31]. A study by Lee et al [32] demonstrated that CHIT1 enhances transforming growth factor-beta (TGF-β) in an animal model of fibrosis, raising the possibility of interactions between CHIT1 and TGF-β also in other diseases, including diabetes. The TGF-β-dependent signaling pathway is for example responsible for podocyte damage in diabetic kidneys [33]. Zhu et al. [34] showed that AMCase induces the production of monocyte chemoattractant protein 1 (MCP-1) and eotaxin-1 in a murine model of asthma, suggesting pro-inflammatory action of this protein. However, it was also demonstrated that AMCase shows an anti-apoptotic effect towards airway epithelial cells and interestingly this action is independent from its chitinolytic activity [35]. These observations indicate that CHIT1 and AMCase are players in cell signaling upon inflammation. In the present study we revealed a positive correlation of AMCase with CHIT1, which may testify that these chitinases cooperate with each other during the inflammatory response. In studies about regulation of AMCase and CHIT1 during macrophages maturation and differentiation, Di Rosa et al. [36] indicated that AMCase, unlike CHIT1, showed increased expression both in classically activated macrophages (inflammatory subtype) and alternatively activated macrophages (anti-inflammatory subtype). It is very likely that expression of these proteins in neutrophils is also regulated by a different mechanism, which should be verified in further studies.

The significant increase of examined proteins in neutrophils of T2D patients, revealed in the present study, suggests that hyperglycemia or other diabetic conditions may promote synthesis of chitinases/CLPs in these cells. It is known that chronic hyperglycemia disturbed some neutrophil functions, but it also exerts a "priming" effect, which is manifested by increased release of various pro-inflammatory cytokines, leading to a constitutively active state and subsequent recruitment of neutrophils and other immune cells. In this way neutrophils participate in the activation and recruitment of macrophages (the main source of GH18 proteins) at the site of inflammation and their further differentiation into proinflammatory or anti-inflammatory subtype [14, 37,38]. In this aspect, content of neutrophilic granules, as well as "excretive" potential of neutrophils, creates an important issue in analyzing participation of these cells in the pathomechanism of type 2 diabetes and its complications. The tendency to increase of GH18 proteins levels in diabetic neutrophils, revealed in the present study, is consistent with our previous investigations in plasma [26,39]. In the present study we considered whether increased values of HbA1c, lipid parameters or blood pressure may result in higher values of GH18 proteins. Most of the patients examined by us, although they received pharmacological treatment, did not achieve the treatment goals set by the Polish Diabetes Association. However, the performed analysis did not reveal any significant differences in the level of examined proteins between subgroups of patients with good and poor metabolic control. It seems that levels of neutrophil-derived CHIT1, AMCase and YKL-40 are independent of achievement of metabolic compensation of diabetes and their increase may be related to other biochemical pathways occurring in diabetes. Moreover, prevalence of CHIT1 mutations did not differ among the different subgroups. However, before concluding that the CHIT1 genotype does not influence the disease severity, studies with more participants should be performed. We also evaluated whether type of received hypoglycemic treatment may influence changes in the levels of GH18 proteins in neutrophils, and we revealed that patients treated with insulin had lower levels of these proteins. However, it is not known if insulin has an in-vivo impact on neutrophil-enzyme expression or degranulation, and the mechanism by which insulin may decrease the level of GH18 proteins remains unknown. We are planning to investigate the influence of insulin on synthesis of these proteins in neutrophils.

We also found that levels of CHIT1, AMCase and YKL-40 were positively correlated with LE and increased progressively with increasing activity of LE. Since LE is considered as a marker of neutrophil activation, our results confirm that activated neutrophils are a source of these proteins in diabetes [40]. Furthermore, increased activity of LE in diabetic neutrophils, as well as its association with poor short-term glycemic control and development of diabetic angiopathies, was previously revealed by us [17]. In fact, LE is able to degrade most components of the extracellular matrix, leading to destruction of the integrity of endothelial cells and damage of the vascular basement membrane. In this light, the relationship we observed of all examined GH18 proteins with LE may indicate their connection with progression of late vascular complications. It is known that diabetic microangiopathies develop as a result of hyperglycemia-induced endothelial dysfunction, which leads to local metabolic and structural disturbances within small vessels and ultimately to vascular damage [41]. We recently demonstrated that increased plasma CHIT1 and YKL-40 are associated with diabetic nephropathy and may participate in the progression of vascular damage within glomeruli in diabetic patients [26]. Here, we revealed that the level of all three examined proteins was the highest in neutrophils of patients with macrovascular complications (being the most significant for CHIT1). As it is known that macroangiopathies in T2D manifest as non-specific atherosclerosis, we suggest that increased levels of GH18 proteins, derived from neutrophils, are connected with inflammatory events triggering development of diabetic vascular complications, especially with an atherosclerotic background. However, it should be mentioned that although levels of all examined proteins were higher in patients with inflammatory evidence, only the concentration of YKL-40 differed significantly between patients differentiated according to CRP concentration. Moreover, despite simultaneous increase of CHIT1 and YKL-40 in diabetic PMNs, we did not reveal any significant correlation between these proteins, which means that their increase was independent, suggesting distinct roles of these two proteins.

Currently it is unknown whether differences in enzymatic activity of GH18 proteins can determine their physiological functions. However, chitinolytic potential of CHIT1 and AMCase leads to the assumptions that, apart from chitin, some still unrevealed targets may exist for human chitinases. Only recently, Larsen et al. [42] showed that N,N'-diacetyllactosamine-terminated saccharides are hydrolyzed by CHIT1. So far, such structures have not been shown in diabetes, but constantly expanding panel of potential substrates for chitinases suggest that other unexpected targets cannot be excluded. For example, it was assumed that GlcNAc containing glycomolecules, such as glycosaminoglycans that are structurally similar with chitin, may be hydrolyzed by chitinases [1]. Negatively charged glycosaminoglycans are present on the endothelial cells surface and their reduced content, observed in diabetic patients, is postulated to be a pathogenic factor for endothelial injury. These could provide an interesting link between neutrophils-endothelial cells interactions and vascular inflammation. However, currently it is not known, if chitinases may be responsible for changes in glycosaminoglycans level and this hypothesis needs to be supported by appropriate research.

In summary, our investigations of "chitinolytic" profile of diabetic PMNs indicate that neutrophil-derived chitinases and chitinase-like proteins represent a novel group of molecules, which may participate in metabolic disturbances and inflammatory pathways in the course of type 2 diabetes, especially connected with development of vascular complications. However, further studies in this area are needed to elucidate molecular cause-and-effect mechanisms of GH18 proteins. Primarily, a potential association of these proteins with markers of endothelial dysfunction should be examined. We also designed ex vivo experiments, in which isolated neutrophils will be subjected to various stimulating factors, and we hope that the results of these investigations will help in finding an answer about the exact molecular pathway responsible for increased levels of GH18 proteins in type 2 diabetes.

## References

[1] Lee CG, Da Silva CA, Dela Cruz CS, Ahangari F, Ma B, Kang MJ, et al Role of chitin and chitinase/chitinase-like proteins in inflammation, tissue remodeling, and injury. Annu Rev Physiol. 2011;73:479–501. 10.1146/annurev-physiol-012110-142250 21054166 PMC3864643

[2] Guo Y, He W, Boer AM, Wevers RA, de Bruijn AM, Groener JE, et al Elevated plasma chitotriosidase activity in various lysosomal storage disorders. J Inherit Metab Dis. 1995;18:717–722. 8750610 10.1007/BF02436762

[3] Bouzas L, Carlos Guinarte J, Carlos Tutor J. Chitotriosidase activity in plasma and mononuclear and polymorphonuclear leukocyte populations. J Clin Lab Anal. 2003;17:271–5. 14614752 10.1002/jcla.10108 PMC6808134

[4] Elias JA, Homer RJ, Hamid Q, Lee CG. Chitinases and chitinase-like proteins in T(H)2 inflammation and asthma. J Allergy Clin Immunol. 2005;116:497–500. 16159614 10.1016/j.jaci.2005.06.028

[5] Johansen JS. Studies on serum YKL-40 as a biomarker in diseases with inflammation, tissue remodelling, fibroses and cancer. Dan Med Bull. 2006;53:172–209. 17087877

[6] Riabov V, Gudima A, Wang N, Mickley A, Orekhov A, Kzhyshkowska J. Role of tumor associated macrophages in tumor angiogenesis and lymphangiogenesis. Front Physiol. 2014;5:75 10.3389/fphys.2014.00075 24634660 PMC3942647

[7] Park SJ, Jun YJ, Kim TH, Jung JY, Hwang GH, Jung KJ, et al Increased expression of YKL-40 in mild and moderate/severe persistent allergic rhinitis and its possible contribution to remodeling of nasal mucosa. Am J Rhinol Allergy. 2013;27:372–80. 10.2500/ajra.2013.27.3941 24119600

[8] Prakash M, Bodas M, Prakash D, Nawani N, Khetmalas M, Mandal A, et al Diverse pathological implications of YKL-40: answers may lie in 'outside-in' signaling. Cell Signal. 2013;25:1567–73. 10.1016/j.cellsig.2013.03.016 23562456

[9] Gu Z, Cao Z, Jin M. Expression and role of acidic mammalian chitinase and eotaxin-3 in chronic rhinosinusitis with nasal polyps. J Otolaryngol Head Neck Surg. 2011;40:64–9. 21303604

[10] Boot RG, van Achterberg TA, van Aken BE, Renkema GH, Jacobs MJ, Aerts JM, et al Strong induction of members of the chitinase family of proteins in atherosclerosis: chitotriosidase and human cartilage gp-39 expressed in lesion macrophages. Arterioscler Thromb Vasc Biol. 1999;19:687–94. 10073974 10.1161/01.atv.19.3.687

[11] Renkema GH, Boot RG, Au FL, Donker-Koopman WE, Strijland A, Muijsers AO, et al Chitotriosidase, a chitinase, and the 39-kDa human cartilage glycoprotein, a chitin-binding lectin, are homologues of family 18 glycosyl hydrolases secreted by human macrophages. Eur J Biochem. 1998; 251:504–9. 9492324 10.1046/j.1432-1327.1998.2510504.x

[12] Esser N, Legrand-Poels S, Piette J, Scheen AJ, Paquot N. Inflammation as a link between obesity, metabolic syndrome and type 2 diabetes. Diabetes Res Clin Pract. 2014;105:141–50. 10.1016/j.diabres.2014.04.006 24798950

[13] Flehmig G, Scholz M, Klöting N, Fasshauer M, Tönjes A, Stumvoll M, et al Identification of adipokine clusters related to parameters of fat mass, insulin sensitivity and inflammation. PLOS One. 2014;9:e99785, 10.1371/journal.pone.0099785 24968098 PMC4072672

[14] Mócsai A. Diverse novel functions of neutrophils in immunity, inflammation, and beyond. J Exp Med. 2013;210:1283–99. 10.1084/jem.20122220 23825232 PMC3698517

[15] Alba-Loureiro TC, Munhoz CD, Martins JO, Cerchiaro GA, Scavone C, Curi R, et al Neutrophil function and metabolism in individuals with diabetes mellitus. Braz J Med Biol Res. 2007;40:1037–44. 17665039 10.1590/s0100-879x2006005000143

[16] Talukdar S, Oh da Y, Bandyopadhyay G, Li D, Xu J, McNelis J, et al Neutrophils mediate insulin resistance in mice fed a high-fat diet through secreted elastase. Nat Med. 2012;9:1407–12. 10.1038/nm.2885 PMC3491143 22863787

[17] Piwowar A, Knapik-Kordecka M, Warwas M. Concentration of leukocyte elastase in plasma and polymorphonuclear neutrophil extracts in type 2 diabetes. Clin Chem Lab Med. 2000;38:1257–61. 11205690 10.1515/CCLM.2000.198

[18] Żurawska-Płaksej E, Piwowar A, Knapik-Kordecka M, Warwas M. Activities of neutrophil membrane-bound proteases in type 2 diabetic patients. Arch Med Res. 2014;45:36–43. 10.1016/j.arcmed.2013.10.003 24316113

[19] Hollak CE, van Weely S, van Oers MH, Aerts JM. Marked elevation of plasma chitotriosidase activity. A novel hallmark of Gaucher disease. J Clin Invest. 1994;93:1288–92. 8132768 10.1172/JCI117084 PMC294082

[20] Boot RG, Blommaart EF, Swart E, Ghauharali-van der Vlugt K, Bijl N, Moe C, et al Identification of a novel acidic mammalian chitinase distinct from chitotriosidase. J Biol Chem. 2001;276:6770–8. 11085997 10.1074/jbc.M009886200

[21] Barrett AJ. Leukocyte elastase. Methods Enzymol. 1981;80:581–8. 7043201 10.1016/s0076-6879(81)80046-8

[22] Boot RG, Renkema GH, Verhoek M, Strijland A, Bliek J, de Meulemeester TM, et al The human chitotriosidase gene. Nature of inherited enzyme deficiency. J Biol Chem. 1998;273:25680–5. 9748235 10.1074/jbc.273.40.25680

[23] Clinical Guidelines of Polish Diabetes Association for the management of diabetes. Diabetol Klin. 2014;3(suppl. A):A1–A71 [in Polish].

[24] Sonmez A, Haymana C, Tapan S, Safer U, Celebi G, Ozturk O, et al Chitotriosidase activity predicts endothelial dysfunction in type-2 diabetes mellitus. Endocrine. 2010;37:455–459. 10.1007/s12020-010-9334-4 20960168

[25] Rathcke CN, Johansen JS, Vestergaard H. YKL-40, a biomarker of inflammation, is elevated in patients with type 2 diabetes and is related to insulin resistance. Inflamm Res. 2006;55:53–9. 16612564 10.1007/s00011-005-0010-8

[26] Żurawska-Płaksej E, Ługowska A, Hetmańczyk K, Knapik-Kordecka M, Adamiec R, Piwowar A. Proteins from the 18 glycosyl hydrolase family are associated with kidney dysfunction in patients with diabetes type 2. Biomarkers. 2015;20:52–7. 10.3109/1354750X.2014.992475 25519006

[27] Volck B, Price PA, Johansen JS, Sørensen O, Benfield TL, Nielsen HJ, et al YKL-40, a mammalian member of the chitinase family, is a matrix protein of specific granules in human neutrophils. Proc Assoc Am Physicians. 1998;110:351–60. 9686683

[28] Cho SJ, Weiden MD, Lee CG. Chitotriosidase in the Pathogenesis of Inflammation, Interstitial Lung Diseases and COPD. Allergy Asthma Immunol Res. 2015;7:14–21. 10.4168/aair.2015.7.1.14 25553258 PMC4274464

[29] Kawada M, Hachiya Y, Arihiro A, Mizoguchi E. Role of mammalian chitinases in inflammatory conditions. Keio J Med. 2007;56:21–7. 17392594 10.2302/kjm.56.21

[30] Kabaroğlu C, Onur E, Barutçuoğlu B, Özhan B, Erdinç S, Var A, et al Inflammatory marker levels in obese adolescents with glucose intolerance: increased chitotriosidase activity. Clin Biochem. 2012;45:281–4. 10.1016/j.clinbiochem.2011.12.007 22206738

[31] Aydogdu A, Tasci I, Tapan S, Sonmez A, Aydogan U, Akbulut H, et al Women with polycystic ovary syndrome have increased plasma chitotriosidase activity: a pathophysiological link between inflammation and impaired insulin sensitivity? Exp Clin Endocrinol Diabetes. 2012;120:261–5. 10.1055/s-0032-1309010 22549343

[32] Lee CG, Herzog EL, Ahangari F, Zhou Y, Gulati M, Lee CM, et al Chitinase 1 is a biomarker for and therapeutic target in scleroderma-associated interstitial lung disease that augments TGF-β1 signaling. J Immunol. 2012;189:2635–44. 10.4049/jimmunol.1201115 22826322 PMC4336775

[33] Zheng X, Bhalla V. The Missing Link: Studying the Alternative TGF-β Pathway Provides a Unifying Theory for Different Components of Diabetic Nephropathy. Diabetes. 2015;64:1898–900. 10.2337/db15-0184 25999532 PMC4439567

[34] Zhu Z, Zheng T, Homer RJ, Kim YK, Chen NY, Cohn L, et al Acidic mammalian chitinase in asthmatic Th2 inflammation and IL-13 pathway activation. Science. 2004;304:1678–82.35. 15192232 10.1126/science.1095336

[35] Hartl D, He CH, Koller B, Da Silva CA, Kobayashi Y, Lee CG, et al Acidic mammalian chitinase regulates epithelial cell apoptosis via a chitinolytic-independent mechanism. J Immunol. 2009;182:5098–106. 10.4049/jimmunol.0803446 19342690 PMC2666938

[36] Di Rosa M, De Gregorio C, Malaguarnera G, Tuttobene M, Biazzo F, Malaguarnera L. Evaluation of AMCase and CHIT-1 expression in monocyte macrophag es lineage. Mol Cell Biochem. 2013;374:73–80. 10.1007/s11010-012-1506-5 23129258

[37] de Vries MA, Alipour A, Klop B, van de Geijn GJ, Janssen HW, Njo TL, et al Glucose-dependent leukocyte activation in patients with type 2 diabetes mellitus, familial combined hyperlipidemia and healthy controls. Metabolism. 2015;64:213–7. 10.1016/j.metabol.2014.10.011 25456098

[38] Espinoza-Jiménez A, Peón AN, Terrazas LI (2012) Alternatively activated macrophages in types 1 and 2 diabetes. Mediators Inflamm. 2012:815953, 10.1155/2012/815953 23326021 PMC3543813

[39] Żurawska-Płaksej E, Rorbach-Dolata A, Knapik-Kordecka M, Piwowar A. Increased chitotriosidase activity in plasma of patients with diabetes type 2. Arch Med Sci. 2016;3 (in press). 10.5114/aoms.2016.60093 PMC5016580 27695487

[40] Viedma Contreras JA. Leucocyte activation markers in clinical practice. Clin Chem Lab Med. 1999;37:607–22. 10475068 10.1515/CCLM.1999.096

[41] Schalkwijk CG, Stehouwer CD. Vascular complications in diabetes mellitus: the role of endothelial dysfunction. Clin Sci (Lond). 2005;109:143–59. 16033329 10.1042/CS20050025

[42] Larsen T, Yoshimura Y, Voldborg BG, Cazzamali G, Bovin NV, Westerlind U, et al Human chitotriosidase CHIT1 cross reacts with mammalian-like substrates. FEBS Lett. 2014; 588, 746–51. 10.1016/j.febslet.2013.12.035 24462685

